# Associations between community participation and types of places visited among persons living with and without dementia: risks perception and socio-demographic aspects

**DOI:** 10.1186/s12877-022-03015-5

**Published:** 2022-04-09

**Authors:** Isabel Margot-Cattin, André Berchtold, Sophie Gaber, Nicolas Kuhne, Louise Nygård, Camilla Malinowsky

**Affiliations:** 1 Department of Occupational Therapy, School of Social Work and Health – HETSL, University of Applied Sciences and Arts of Western Switzerland (HES-SO), Lausanne, Switzerland; 2 Division of Occupational Therapy, Department of Neurobiology, Care Sciences and Society (NVS), Karolinska Institutet, Stockholm, Sweden; 3 Institute of Social Sciences, University of Lausanne, Lausanne, Switzerland

**Keywords:** Community participation, Dementia, Risks perception, Places visited

## Abstract

**Introduction:**

Increasingly, literature has focused on community participation in places visited by persons living with and without dementia. Earlier research indicates that multiple factors, including socio-demographic aspects and risk perception may influence community participation.

**Aim and methods:**

This cross-sectional, explorative study aims to inquire into how places visited, socio-demographic aspects and risks perception are associated with self-rated community participation for persons living with and without dementia (*n* = 70) in Switzerland. Data was collected through face-to-face interviews with questionnaires (ACT-OUT, MoCA, sociodemographic). First, we investigated whether the number of places visited was correlated with self-rated participation; then we added socio-demographic and risks perception factors with a bivariate analysis; and searched for a model using multinomial logistic regressions.

**Results:**

For the group of participants living with dementia, risks of falling (*p* = .014) and of getting lost (*p* = .037) were significantly associated with self-rated participation. For the group of participants living without dementia, visiting places outside the home was significantly associated with self-rated participation, especially visiting places in domain D/places for recreational and physical activities (*p* = .005).

**Discussion and conclusions:**

The results of exploring multiple factors and searching for a model highlights the complexity of community participation as a construct. Risks and visiting places for recreational and physical activities seem to play a role in self-rated participation. Mobile interviews might be better suited to gain in-depth understanding on community participation for persons living with dementia.

## Introduction

Dementia is a condition which includes progressive memory impairment and loss of other cognitive functions [1, 2]. Cognitive decline in dementia and its impact on basic and instrumental activities of daily living (ADL(IADL) has been widely and thoroughly examined [3–6]; however, participation and engagement in activities [7, 8], as well as community participation have been studied to a lesser degree [9, 10]. Community participation is understood here as the engagement in meaningful activities performed in places outside the home, also referred to as out-of-home participation. As such, community participation is contextualised, situated and embedded in places visited by people, underscoring the person-environment relationship experienced while engaging in activities [11–13]. Given that the experience of community participation is situated, there is a need to increase our knowledge on the impact of visiting diverse and numerous places.

Places are understood here as being relational [14, 15]. Through the regular performance of meaningful activities in spaces located outside the home, people create familiar places, where they feel they belong, have control over their lives and can maintain community participation. This constant relationship, or transaction, [16] with the environment through the activities performed there enables people and especially persons living with dementia to experience a sense of familiarity and belonging toward the places they visit [15, 17]. In recent years, there has been an increasing volume of literature on out-of-home participation, including a focus on the neighbourhood as being of importance for persons living with dementia [12, 15, 18]. A series of studies have been conducted on out-of-home participation focusing on places visited by persons living with dementia in Canada, Sweden, Switzerland and the United Kingdom. The results of these studies have shown that persons living with dementia are likely to experience not just a global decrease of their community participation, but rather a shift from visiting social and cultural places to places used for medical and self-care [19]. Based on comparisons of the number of places visited in the past and present, persons living with dementia have also been shown to experience significantly more abandonment of places than their peers without dementia [20]. The studies mentioned above are based on the assumption that visiting places is partly an expression of out-of-home participation [21–23]. However, as persons living with dementia experience a shrinking world in terms of quantity and diversity of places visited, understanding the quality of the relationship they maintain with a few meaningful places becomes of utmost importance [13]. There is an assumption that the more diverse range of places people visit, the better they rate their community participation. The first aim (1) of this study was to question this assumption and to discuss it.

Additionally, socio-demographic factors have been highlighted as influencing out-of-home participation and how it is perceived by persons living with dementia, showing that a multitude of factors may have an impact [24]. These include intrapersonal factors like age, gender and education level; interpersonal factors like living alone or with someone [25]; and environmental factors like living in a rural or urban context [26]. Availability of amenities, transportation, and driving issues have been found to have an impact on community participation [27, 28].

Limiting factors for out-of-home participation include old age, female gender, low socioeconomic status, social deprivation of the living environment, comorbidity, lifestyle, lack of motivation, weak social network and limited social activities [29, 30]. Being a driver [31] or losing access to a car [28] are factors or events that also have an impact on community participation. Having a diagnosis of dementia adds limiting factors [9], especially considering the level of stigmatisation persons living with dementia are subjected to [32] and underscores the complexity of understanding what influences community participation.

Furthermore, how risks are constructed and perceived have been identified as playing a role in community participation and dementia care [33, 34]. Research on risks has mostly addressed health consequences, safety issues and prevention of the event identified as a risk [35]. Persons living with dementia may experience family, friends and service providers framing risks as “unwanted”, “undesirable” or “to be avoided”. This construction of risks is based on the importance given to safety in dementia care. As a result, older adults with dementia have reported reducing their involvement in activities outside the home and confining themselves to home as a way of increasing safety [36, 37]. This construction of risks focuses on a depiction of risk as negative or dangerous, stressing the potential loss; instead of a more nuanced concept that can bring “gains” to the person living with dementia [38, 39]. There are positive aspects of risk taking, such as expressing one’s individuality, independence and control over one’s life; which could reframe activities outside the home that appear hazardous, into activities that are perceived as positive and identity enhancing [40, 41]. A more nuanced approach to risk in dementia studies might broaden the experience of community participation to include feelings of independence, freedom, and control.

Still, getting lost is a commonly perceived risk by persons living with dementia and their families. It is often associated with wandering behaviours and used as a reason to limit community participation [42, 43]. Falling is another commonly perceived risk, by both persons living with and without dementia, and fear of falling is an aggravating factor. Research suggests that approximately half of all falls occur in the street or in places outside home [44]. Feeling stressed or embarrassed are also identified as a risk by the persons living with dementia [45]. Despite this, little research has examined how the way persons perceive these risks might be associated with the self-rating of their community participation.

A study on risk construction and perception conducted in Switzerland with triads of persons living with dementia, their significant others and home-health professionals, has shown that risks need to be understood as dynamic and co-constructed [46]. Coping strategies are often used by persons living with dementia to manage situations fraught with risks, such as staying at home and withdrawing from out-of-home activities, asking for help to be driven to appointments, relying on significant others to go out. For some persons living with dementia, coping strategies include simply being aware of risks, tolerating them when going out, and even experiencing them as challenges to be overcome [47]. How risks are seen, constructed, and experienced may shape how persons living with dementia participate outside their home, offering a rationale for examining the relationship between their perception of risks and community participation.

In summary, it is hypothesised that the number of places visited outside the home might have an influence on how older adults rate their community participation. As out-of-home participation is a complex, dynamic, and multi-faceted phenomenon, other factors than visiting places may influence how persons living with and without dementia experience and perceive it. Such additional factors are included in socio-demographic variables and perceived risks. To our knowledge, these factors have not yet been investigated in relation to community participation.

The second aim (2) of this study was to explore how these factors were associated with the self-rating of community participation for a sample of persons living with and without dementia in Switzerland. This study addressed the following research questions:

1. What associations existed between the number of places visited and self-rated community participation among a sample of persons living with and without dementia?
2. What factors (e.g., amount and types of places visited, socio-demographic variables and perceived risks) might have been associated with the self-rating of community participation?

## Methods

### Design and setting

This cross-sectional, explorative study reports on research undertaken within the “Life outside home for people with dementia” (OUTDEM) setting as part of a larger project led by Karolinska Institutet in Sweden. Standardised questionnaires were used in interviews with participants living with and without dementia in both rural and urban regions of the French-speaking region of Switzerland.

### Participants and recruitment

All participants (*n* = 70) were community-dwelling older adults (aged > 65 years old). Recruitment started in December 2015 and ended in May 2017. Participants with dementia (*n* = 35) were recruited through memory clinics, day hospitals, and the Swiss Alzheimer’s Association. Diagnosis of dementia was established by physicians at memory clinics. Participants in the comparison group (*n* = 35) were recruited through senior associations and advertisements in grocery stores. We aimed to match, but not pair, the comparison group of persons living with no known cognitive impairment (*n* = 35) with the dementia group regarding age, gender, education level, living areas and settings. Thus, recruitment strategies for the comparison group targeted specific regions, age groups, or living areas, for example, to bring the distribution of the comparison group closer to the dementia group on those variables.

An approximate required sample size was calculated based on the difference of the total number of places visited between the 26 older adults and the five persons living with dementia who took part in an earlier study presenting the development of the Participation in ACTivities and Places OUTside Home Questionnaire (ACT-OUT) [48]. The same approximate sample size of groups of 35 have been used in similar studies [20, 49]. No formal power calculation was conducted due to the exploratory design. The results from this study might be used with the findings from earlier studies to guide power calculations for future research using the ACT-OUT.

### Data collection procedures

The interviews were conducted by two registered occupational therapists, one of whom was the first author. Both interviewers had prior knowledge of using the ACT-OUT and they had harmonised with each other the way they conducted the interviews [50]. The interviews were comprised of three standardised questionnaires, performed in the following order: (i) the Participation in ACTivities and Places OUTside Home Questionnaire (ACT-OUT) [48]; (ii) the Montreal Cognitive Assessment (MoCA) [51]; and (iii) a socio-demographic questionnaire. Written and verbal informed consent was obtained from each participant prior to data collection. The “process consent method” proposed by Dewing (2002) [52] was used in this study because it is person-centred, and it enables researchers to include consent communicated through behaviours and non-verbal means by the person with dementia. Following Dewing (2007) [53], ongoing consent monitoring was implemented throughout the data collection process to ensure no stress or burden from participating in the project occurred [54]. To mitigate against fatigue and potential burden, interviews occurred in the participant’s home and were adapted to each participant e.g., inviting a significant other for emotional support, or spreading the interviews across sessions lasting no more than 2 h. Ethical approval (protocol 452/15) was obtained from the “Commission cantonale d’éthique de la recherche sur l’être humain (CER-VD)” in Lausanne, Switzerland.

### Data analysis: questionnaires and variables

The ACT-OUT has three parts. Part I includes a list of 25 pre-determined types of places, grouped into four domains: A/ consumer, administrative, and self-care places (*n* = 7); B/ places for medical care (*n* = 5); C/ social, cultural, and spiritual places (*n* = 6); and D/ places for recreational and physical activities (*n* = 7). The interview uses Part I to ask questions about whether respondents visit these places in the past, present, and future. Using Part II, the interviewer poses detailed questions about factors potentially influencing participation in places retained and abandoned, such as the types of activities performed, means of transportation, the presence of accompanying persons, risk perception, and familiarity. Part III consists of general questions about perceived out-of-home participation, life satisfaction, and attitudes towards risk-taking and stress factors.

The Montreal Cognitive Assessment (MoCA) [51] was used as a comprehensive screening tool for assessing and describing the level of cognitive functioning for both groups. It consists of sections focusing on diverse cognitive functions (e.g., memory, time and space orientation, visual perception). The total MoCA score reflects the cognitive level of each participant.

A socio-demographic questionnaire was used to collect data regarding age, gender, education, living arrangement, setting (urban/rural), and access to using a car.

For the purposes of this study, data from the ACT-OUT (Part I and III), the MoCA total score and socio-demographic questions were used.

### Dependant variable: self-rated participation

The dependent variable was the data collected by the question from Part III of the ACT-OUT (*How do you perceive your participation in all situations outside home to be?*), to which participants responded with (4 = I participate as I wish; 3 = I participate almost as I wish; 2 = I rather do not participate as I wish; 1 = I do not participate as I wish). Only one participant with dementia responded, “I do not participate as I wish”, the lowest level, thus allowing the aggregation of levels 1 and 2.

### Independent variables

The fifteen independent variables were derived from the ACT-OUT questionnaire (Part I: number of places visited (*n* = 5) and Part III: risks (*n* = 4)) and socio-demographic questionnaire (*n* = 6); and are described in Table 1.

**Table 1:** Presenting 15 independent variables used to search for associations with self-rated community participation

***Number of visited places by domain (ACT-OUT)***	
Total number of currently visited places (max =25)	
Number of places visited in domain A/ consumer, administrative and self-care places (max = 7)	
Number of places visited in domain B/places for medical care (max = 5)	
Number of places visited in domain C/social, cultural and spiritual places (max = 6)	
Number of places visited in domain D/places for recreational and physical activities (max = 7)	
***Characteristics of the participants (MoCA and socio-demographic questionnaire)***	
Age expressed in years	
Gender expressed as male or female	
Education was adapted into three levels from The International Standard Classification of Education (ISCED 2011) [55] (primary/secondary school, apprenticeship, and higher education degree)	
Living arrangement expressed as living alone or with someone	
Setting adapted to the Swiss context as village, small town and city	
Access to using a car expressed as self-driving, being driven by someone or no use of car	
***Perceived risks (ACT-OUT)***	
Getting lost expressed as concern perceived by participants (*very concerned*; *concerned*; *unconcerned*; *very unconcerned)*	
Falling down	
Being stressed	
Being embarrassed	

The four types of perceived risks were identified in earlier research about persons living with dementia [45, 47] and included: (i) falling; (ii) getting lost; (iii) feeling stressed; and (iv) feeling embarrassed. No participants responded “very concerned*”* about any of the risks and thus, the “very concerned” was not included.

### Statistical analyses

First, we computed descriptive statistics for all variables and we systematically tested the differences between the dementia and comparison groups using the Fisher exact test for categorical variables, and the t-test for continuous variables. To explore the associations between the total number of places and the self-rated community participation, Spearman correlations coefficients were evaluated for the dementia group and the comparison group separately, as well as for the full group of 70 participants. The strength of association was classified using Cohen’s guidelines for social sciences: .1–.3 (small); .3–.5 (medium); and .5–1.0 (large) [56]. To test the differences between the total number of places by the level of self-rated community participation, we used the Mann-Whitney U test for the comparison group (two levels of self-rated participation) and the Kruskal-Wallis test for the dementia group (three levels of self-rated participation). We used non-parametric tests as the data was not normally distributed and due to the small sample size.

Second, we used bivariate regressions to explore the associations between each of our independent variables and self-rated community participation. As the group variable was significant (*p* < .001) in the full sample analyses, we performed the regressions for the dementia and comparison groups separately. We used logistic regressions for the comparison group, since the dependent variables had two categories, and multinomial regressions for the dementia group, since the dependent variable had three categories. At this point, we selected all independent variables that were significant at the bivariate level, and we entered them together into multivariate regressions.

For all regressions, we used the highest level of self-rated participation as the reference category. The Type I error was set to .05 for all analyses. Results are presented as relative risk ratios (RRR) with 95% confidence intervals for the multinomial regressions, and as odds ratio (OR) with 95% confidence intervals for the logistic regressions. We performed all analyses in the Statistical Package for Social Sciences (SPSS) computer software, version 25.

## Results

The groups of participants were closely matched regarding most of the socio-demographic variables, including age, gender, educational levels or rural/urban settings. The group of participants living with dementia had significantly lower scores in cognitive functioning as described by the MoCA (*p* = <.001), significantly lower access to a car (*p* = <.001), visited significantly fewer places outside their home (*p* = <.001), and perceived significantly more risks, like getting lost (*p* = <.001), compared to the group of participants living without dementia. The group of participants living with dementia also rated their community participation as significantly lower (*p* = <.001) than the comparison group (See Table 2).

**Table 2:** Description of the outcome (dependent) variable and independent variables. For the comparison test, we used the Fisher exact test for categorical variables, and the t-test for continuous variables

Variables	Dementia	Comparison	CI 95% of mean difference	Coefficient for the difference between groups	*p*-value
	***n*** = 35	***n*** = 35			
**Outcome: Participation (ACT-OUT, Part III)**				**18.740**	**<.001**
Participate only a little	12	0			
Do participate	8	6			
Participate as much as wanted	15	29			
***Number of visited places by domain (ACT-OUT)***					
Nb of currently visited places (ACT-OUT, max = 25)				3.893	<.001
Mean (SD)	15.83 (3.34)	18.91 (3.28)	1.50; 4.66		
Nb visited places – Domain A (max = 7)				4.622	<.001
Mean (SD)	4.43 (2.11)	6.31 (1.15)	1.07; 2.70		
Nb visited places – Domain B (max = 5)				−2.653	.010
Mean (SD)	3.26 (0.98)	2.71 (0.71)	−.95; −.13		
Nb visited places – Domain C (max = 6)				3.144	.002
Mean (SD)	3.71 (1.25)	4.66 (1.25)	.34; 1.54		
Nb visited places – Domain D (max = 7)				2.661	.010
Mean (SD)	4.11 (1.18)	5.03 (1.65)	.23; 1.60		
***Characteristics of the participants (MoCA and socio-demographic questionnaire)***					
**MoCA**				8.195	<.001
Mean (SD)	17.74 (5.56)	26.09 (2.07)	6.31; 10.37		
**Age**				.104	.917
Mean (SD)	77.66 (8.35)	77.86 (7.72)	−3.63; 4.03		
**Gender**				.952	.333
Male	16	12			
Female	19	23			
**Education**				.110	.950
Mandatory school	9	10			
Apprenticeship	17	17			
Higher education degree	9	8			
**Living arrangement**				.560	.618
Alone	11	14			
Living with someone	24	21			
**Setting**				2.142	.384
Rural	15	14			
Small town	14	10			
City	6	11			
**Access to using a car**				13.984	.001
Not a car user	11	7			
Car user (self-driving)	7	22			
Car user (someone else drives)	17	6			
***Perceived risks (ACT-OUT)***					
**Getting lost**				21.167	<.001
Concerned	11	0			
Unconcerned	7	1			
Very unconcerned	17	34			
**Falling down**				2.146	.350
Concerned	7	3			
Unconcerned	8	11			
Very unconcerned	20	21			
**Being stressed**				9.762	.008
Concerned	10	2			
Unconcerned	10	6			
Very unconcerned	15	27			
**Being embarrassed**				11.427	.003
Concerned	11	2			
Unconcerned	7	3			
Very unconcerned	17	30			

### Association between the number of places visited and self-rated participation

**Fig. 1:**
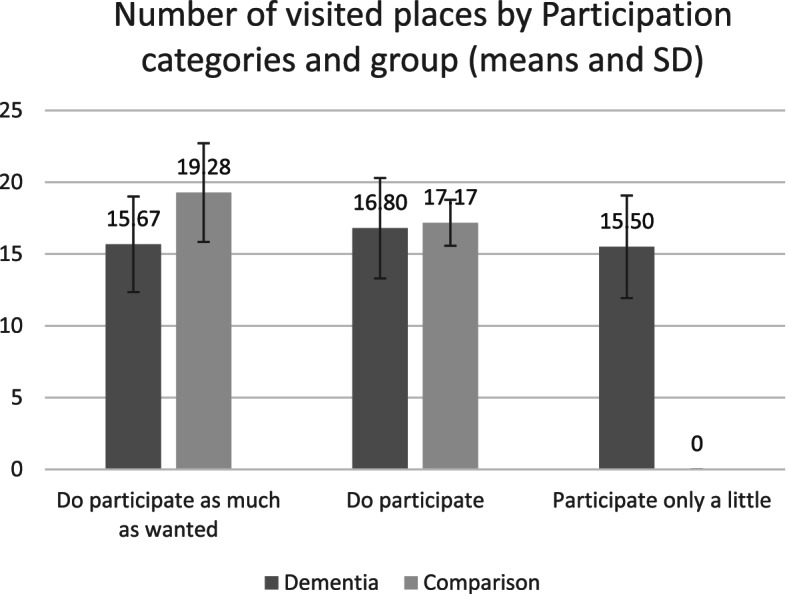
Mean number of places visited per level of self-rated participation and per group

Among the entire group, we found a significant association (*p* = .036) between self-rated participation and the total number of places visited. The strength of the association was small (r_s_ = .251) and positive, meaning that the more places that a person visited, the better they rated their community participation. The comparison group showed a significant correlation (*p* = .043) between self-rated participation and the total number of places visited, and the strength was medium (r_s_ = .344) and positive. However, in the group of participants living with dementia, the correlation was not significant. Figure 1 illustrates the mean number of places visited in relation to the participation rated by each group. When looking at the mean of the number of places visited by participants based on their self-reported participation, it shows that the comparison group had a significant decrease of the mean of places visited (*p* = .044), compared to the dementia group which shows non-significant fluctuations (See Fig. 1). These results may indicate that the number of places is not the deciding factor for the group of participants living with dementia to rate their community participation.

### Exploration of associations between self-rated community participation and multiple variables

Although the education variable has been identified as a factor influencing out-of-home participation in earlier research, it proved to be problematic in our samples, since there were not enough participants in each category to calculate relative risk ratios for all three groups. Thus, we decided to remove the education variable from the bivariate regressions. Most of the variables proved to be not significant at the bivariate level, for either group samples. The results in the bivariate analysis may indicate that only a few factors were significantly associated with self-rated participation.

Table 3 presents the results for the group of participants living with dementia. Only the risk variables of getting lost (*p* = .037) and falling (*p* = .014) were significantly associated in the bivariate multinomial regressions and were included in the multivariate regression. In the multivariate regression, no independent variable was significant. Both getting lost and falling were reported as hindering the self-rated community participation. These results may indicate that few personal socio-demographic variables play a role in the rating of community participation and that participants living with dementia recognise some risks but might not be influenced by the number of places visited when rating their community participation.

**Table 3:** Bivariate and multivariate multinomial regressions for the level of participation for the dementia group, using “participate as I wish” as the reference category

**Group living with dementia: Bivariate (each variable treated separately)**								
Independent variable	Participate only a little (2)			Do participate (3)			Nagelkerke R2	*p*-value
	RRR	CI 95%	*p*-value	RRR	CI 95%	*p*-value		
Risks								
Getting lost (ref: very unconcerned (4))							.287	**.037**
Concerned (2)	4.20	.59–30.09	.153	2.10	.25–17.59	.494		
Unconcerned (3)	.23	.02–2.59	.236	(a)	(a)	(a)		
Falling down (ref: very unconcerned (4))							.340	**.014**
Concerned (2)	(a)	(a)	(a)	(a)	(a)	(a)		
Unconcerned (3)	1.38	.18–10.65	.760	1.10	.15–8.13	.926		
Stress (ref: very unconcerned (4))							.033	.904
Concerned (2)	.84	.13–5.26	.852	.93	.11–7.82	.949		
Unconcerned (3)	1.87	.28–12.31	.517	2.33	.29–18.97	.428		
Embarrassment (ref: very unconcerned (4))							.111	.462
Concerned (2)	1.94	.32–11.76	.469	1.75	.23–13.16	.587		
Unconcerned (3)	.23	.02–2.59	.236	.35	.03–4.15	.406		
Socio-demographic								
Age	1.00	.91–1.09	.996	.99	.90–1.10	.921	.000	.994
Gender (ref: female (2))	.48	.10–2.23	.346	.22	.03–1.50	.122	.086	.250
Living situation (ref: with someone (2))	.50	.10–2.65	.415	.50	.07–3.36	.476	.028	.641
Access to car (ref: someone else drives (2))							.031	.914
Not a car user (0)	1.12	.19–6.41	.899	.56	.08–4.14	.570		
Car user – self-driving (1)	1.40	.19–10.03	.738	.47	.04–5.90	.467		
Setting (ref: big city (2))							.136	.345
Rural (0)	1.00	.13–7.57	1.00	(a)	(a)	(a)		
Small town (1)	.57	.08–4.29	.587	(a)	(a)	(a)		
Places visited								
Total number of places visited	1.08	.85–1.37	.510	.95	.74–1.23	.710	.029	.635
Total places visited in A	1.00	.70–1.43	1.00	1.11	.72–1.70	.647	.008	.881
Total places visited in B	1.49	.67–3.33	.334	.99	.40–2.47	.984	.037	.561
Total places visited in C	.88	.46–1.71	.710	.49	.22–1.09	.079	.115	.155
Total places visited in D	1.87	.83–4.22	.133	.67	.30–1.48	.316	.167	.062
**Group living with dementia: Multivariate (Nagelkerke R2 .468,** ***p*** **= .017)**								
Independent variable	Participate only a little (2)			Do participate (3)			*p*-value	
	OR	CI 95%	*p*-value	OR	CI 95%	*p*-value		
Getting lost (ref: very unconcerned (4))							.187	
Concerned (2)	1.71	.15–19.19	.663	1.70	.18–16.46	.644		
Unconcerned (3)	.35	.03–4.20	.406	(a)	(a)	(a)		
Falling down (ref: very unconcerned (4))							.077	
Concerned (2)	(a)	(a)	(a)	(a)	(a)	(a)		
Unconcerned (3)	1.24	.14–11.06	.848	.98	.10–9.58	.988		

^a^Not enough participants in this category to get results

Table 4 shows the results from the set of bivariate multinomial regressions for the comparison group. The results for the risk variables show that most participants in the comparison group perceived risks as unrelated to their rating of community participation. Only the total number of places visited in domain D (*p* = .005) was significantly associated with self-rated participation. Since only one predictor was significant for the comparison group at the bivariate level, no additional regression was required. These results indicate that visiting places related to recreational and physical activities (domain D) may increase the rating of community participation for the comparison group of participants living without dementia. This comparison group did not seem to link their rating of community participation to perceived risks.

**Table 4:** Bivariate logistic regressions for the level of participation for the comparison group, using “participate as much as I want” as the reference category and each independent variable treated separately

Comparison group					
Independent variable	Do participate (3)			Nagelkerke R2	*p*-value
	OR	CI 95%	*p*-value		
Risks					
Getting lost (ref: very unconcerned (4))				(a)	(a)
Unconcerned (3)	(a)	(a)	(a)		
Falling down (ref: very unconcerned (4))				.199	.109
Concerned (2)	(a)	(a)	(a)		
Unconcerned (3)	5.43	.81–36.51	.082		
Stress (ref: very unconcerned (4))				.218	.086
Concerned (2)	(a)	(a)	(a)		
Unconcerned (3)	8.00	1.08–59.14	.042		
Embarrassment (ref: very unconcerned (4))				.057	.544
Concerned (2)	(a)	(a)	(a)		
Unconcerned (3)	2.50	.19–33.17	.487		
Socio-demographic					
Age	1.08	.96–1.23	.212	.080	.191
Gender (ref: female (2))	2.22	.37–13.22	.380	.036	.383
Living situation (ref: with someone (2))	.25	.03–2.38	.246	.084	.178
Access to car (ref: someone else drives (2))				.003	.971
Not a car user (0)	.83	.04–16.99	.906		
Car user – self-driving (1)	1.11	.10–12.31	.932		
Setting (ref: big city (2))				.038	.666
Rural (0)	2.73	.24–30.66	.416		
Small town (1)	2.50	.19–32.80	.485		
Places visited					
Total number of places visited	.83	.64–1.08	.172	.090	.164
Total places visited in A	.79	.41–1.51	.476	.022	.493
Total places visited in B	2.24	.52–9.72	.281	.061	.252
Total places visited in C	1.34	.62–2.91	.462	.027	.446
Total places visited in D	.44	.23–.85	.015	.331	**.005**

^a^Not enough participants in this category to get results

## Discussion

The aim of the study was to explore how various factors were associated with the self-rating of community participation for a sample of persons living with and without dementia in Switzerland. Our results suggest that living with dementia is a significant factor that seems to change not only the number and types of places visited, but also community participation. This result is aligned with earlier research using the ACT-OUT [19, 20], which revealed that having a dementia diagnosis is likely to impact the way that persons access, visit and use out-of-home places. Thus, the results from this present study and earlier research challenge the assumption that the number of places visited is associated with community participation for persons living with dementia.

### Association between the number of places visited and self-rated community participation

In response to aim 1, the results showed that for the comparison group, the number and diversity of places visited seems to be associated with self-rated participation. It is possible to infer that the more the persons living without dementia visit a diversity of places, the more they perceive their community participation to be satisfactory for themselves. However, for the group living with dementia, there was no such association. Research indicates that persons living with dementia tend to become more dependent on a significant other, and this may influence how, when, and where they go outside as well as what types of activities they engage in [57]. Out-of-home participation may be influenced by the persons living with dementia in relation to their significant others, in a way that might be described as co-occupations [58]. Co-occupations are performed in interdependence with others and offer a specific theoretical lens for looking at participation, involving significant others. However, persons living with dementia may also resign from social activities and abandon places related to these activities. Similarly, as persons living with dementia reduce going to these specific places, the places might lose the meaning that formerly had been attributed to them [59]. Furthermore, places may become less familiar as the person’s disease progresses [19].

However, by contextualising the experience of community participation, for instance in relation to the availability of support or interventions to address barriers such as social exclusion and stigmatisation, it might refine our understanding of the difficulties and resources persons living with dementia have in their communities [32]. The ways in which persons living with dementia are seen, perceived and how their behaviours are judged may also be linked to the un/availability of befriending others in out-of-home places [60]. This study’s results suggest that there is a need for a better inclusion of situational and contextual factors as experienced by persons living with dementia in their communities, as these have the potential to influence the range of places visited. More specific research on places, including perceived meaning and sense of belonging of places, as well as support for engagement in community participation, is needed to better understand the complex relationship between the person living with dementia and their environment.

### Exploration of associations between community participation and multiple variables

In response to aim (2), the results on the exploration of multiple variables revealed few significant associations with self-rated community participation. This may be partially due to the small samples in this study and the education variable had to be removed from the analyses as there were an insufficient number of participants in some categories. Still, places especially in domain D/places for recreational and physical activities, were associated with self-rated participation for the comparison group, but not the group of persons living with dementia. However, for the latter group, places visited in domain D was the closest of all types of places to being significant (*p* = .062). Domain D contains a range of places that are linked to physical activity, contact with nature, travelling and vacation, and the neighbourhood. Hence, it would be reasonable to expect an association between visiting these places, especially the neighbourhood [13] and out-of-home participation. Physical activity has been shown to positively influence cognitive functioning, quality of sleep and perception of self, and to prevent depression [61, 62]. Contact with nature offers a restorative effect linked with greenery that increases positive emotions, reduces anger, and helps heighten self-awareness [63, 64]. Older adults have also reported that travelling is important to them as it has been shown to prevent disease, and to maintain high levels of physical and cognitive function; travelling is also associated with better engagement in community participation [28, 65, 66]. In earlier research, the neighbourhood has been shown as a place to support social relations, engagement in life, and community participation [12, 15, 18]. Some of the places in domain D may be located near to the person’s home, such as the garden, or the neighbourhood. However, other places in domain D may be located at a further distance from the person’s home, such as a summer house or a train station for travelling. In earlier studies, the abandonment rate of visiting a summer house or cottage was shown as being above 50% for the group of persons living with dementia and above 20% for the comparison group [19, 20]. As domain D includes various places that each could have a potential association with community participation, further research is needed to determine how each place in this domain might be perceived by persons living with and without dementia.

It is interesting to note that no socio-demographic variable was identified as showing a significant association with self-rated community participation in our results. As these variables are usually highlighted in research [24, 67] for having an impact on well-being and participation, the results of this study show a contradiction with earlier research. The results in this study point towards a more non-linear, complex and multifactorial understanding of community participation, as has also been stressed in earlier research [9]. The experience of community participation being situated and embedded in places that are mostly familiar [17] also questions the emphasis given to socio-demographic factors like age, gender, education, and settings in dementia research, when availability of commodities, health services and enabling interventions may also play a role. This should encourage us to design more relational and in-depth research on community participation. Furthermore, the lack of significant association in the results might also be due to the small sample size.

Two (getting lost and falling) of the four risk variables included in this study were significantly associated with self-rated community participation, among the group of persons living with dementia. However, given that neither the risk of getting lost nor falling were significant in the search for a (multivariate) model for that group, it seems that participants who perceive getting lost as a risk also perceive falling as one. Perceiving space outside the home as fraught with risks [35] might be what is associated with self-rated community participation, instead of specific risks. There is a need to better understand how risks are perceived by persons living with dementia and their family [46]; and how they are socially constructed in the local context [36, 45] in terms of social bias and stigmatisation. Although risks are often seen as having a restricting influence on community participation, especially for persons living with dementia [36], some authors have argued that risks could be perceived as positive, offering challenges that will “add a spark” to life [39]. Supporting a more positive representation of risks when related to community participation of older adults [68, 69], and especially those living with dementia, might also increase their opportunities to maintain visiting important and meaningful places; and incidentally address the stigmatisation of having dementia in our society by recognising continued citizenship [32, 70].

### Study limitations

The small sample size is a limitation of this study that warrants attention. Although 70 participants were included in the entire sample, the factor of living with a dementia was shown to be significant in the analysis, supporting the idea of conducting the exploration of multiple factors separately for both groups. As the two groups were comprised of small samples, we first used bivariate analysis before looking for a model to mitigate the effect of having a small sample size. Looking for a model with small samples is tentative, as the number of factors and steps in the scales adds to the number of degrees of freedom and might make the results less reliable. Therefore, our results need to be considered with respect to this limitation. Still, this exploratory study provides insights into community participation for persons living with dementia.

Another limitation of this study is that we did not collect data on characteristics of the participants’ community, such as the availability of amenities, commodities, transportation, community support, dementia-friendly initiatives, or community interventions, which could potentially have been included in the factors under exploration. It is uncertain how the absence of these variables might have changed our results. In addition to socio-demographic variables, self-rated community participation is dependent on individual preferences as well as contextual availability and support, making this construct very complex to apprehend. There is a need to better specify the impact of socio-demographic and contextual factors and include individual preferences on community participation in future longitudinal studies for persons living with dementia.

Collecting data with persons living with dementia through the use of face-to face interviews raises the question of the reliability of the data [71, 72], especially to rely on episodic memory to narrate autobiographical facts. These types of interviews offer only a “snap-shot” in time and in a static manner. Thus, using mobile or walking interviews might be better suited to collect data on community participation [17], which is a non-linear, complex, contextualised and situated experience, especially for persons living with dementia.

## Conclusion

We found that community participation was associated with the number of places visited but only for the comparison group of persons living without dementia. Our exploration of multiple factors and search for a model, highlights the complexity and situatedness of community participation as a construct. Visiting many diverse types of places seemed to play a role in participation outside home and future research would benefit from exploring this using a variety of methods, including qualitative research and mobile interviews.

## Data Availability

The datasets generated and/or analysed during the current study are not publicly available due to ongoing international secondary analysis but are available from the first author on reasonable request (Isabel.margot@hetsl.ch). The data (from Switzerland) used in this study has also been shared within the ACT-OUT development project under a data sharing convention and funding from the Kamprad Family Foundation. The larger dataset comprises data from 4 countries (Sweden, Switzerland, the United Kingdom, and Canada) and is currently being analysed in comparison to each country. Access to the combined dataset may also be granted upon reasonable request, from the first author (Isabel.margot@hetsl.ch).
